# Longitudinal disease-associated gut microbiome differences in infants with food protein-induced allergic proctocolitis

**DOI:** 10.1186/s40168-022-01322-y

**Published:** 2022-09-23

**Authors:** Victoria M. Martin, Yamini V. Virkud, Ehud Dahan, Hannah L. Seay, Dvir Itzkovits, Hera Vlamakis, Ramnik Xavier, Wayne G. Shreffler, Qian Yuan, Moran Yassour

**Affiliations:** 1 Food Allergy Center, Massachusetts General Hospital, Boston, MA USA; 2 Division of Pediatric Gastroenterology, Hepatology and Nutrition, Massachusetts General Hospital, Boston, MA USA; 3 Department of Pediatrics, Harvard Medical School, Boston, MA USA; 4 Food Allergy Science Initiative of the Broad Institute, Cambridge, MA USA; 5 Division of Pediatric Allergy and Immunology, Massachusetts General Hospital, Boston, MA USA; 6 The Rachel and Selim Benin School of Computer Science and Engineering, The Hebrew University of Jerusalem, Jerusalem, Israel; 7 Microbiome and Infectious Diseases, The Broad Institute of MIT and Harvard University, Cambridge, MA USA; 8 Center for Computational and Integrative Biology, Massachusetts General Hospital, Boston, MA USA; 9 Department of Molecular Biology, Massachusetts General Hospital, Boston, MA, USA; 10 Klarman Cell Observatory, Broad Institute of MIT and Harvard, Cambridge, MA USA; 11 Pediatrics at Newton Wellesley, P.C, Newton, MA USA; 12 Microbiology & Molecular Genetics Department, Faculty of Medicine, The Hebrew University of Jerusalem, Jerusalem, Israel

## Abstract

**Background:**

Complex interactions between the gut microbiome and immune cells in infancy are thought to be part of the pathogenesis for the marked rise in pediatric allergic diseases, particularly food allergies. Food protein-induced allergic proctocolitis (FPIAP) is commonly the earliest recognized non-immunoglobulin E (IgE)-mediated food allergy in infancy and is associated with atopic dermatitis and subsequent IgE-mediated food allergy later in childhood. Yet, a large prospective longitudinal study of the microbiome of infants with FPIAP, including samples prior to symptom onset, has not been done.

**Results:**

Here, we analyzed 954 longitudinal samples from 160 infants in a nested case-control study (81 who developed FPIAP and 79 matched controls) from 1 week to 1 year of age by 16S rRNA ribosomal gene sequencing as part of the Gastrointestinal Microbiome and Allergic Proctocolitis (GMAP) study. We found key differences in the microbiome of infants with FPIAP, most strongly a higher abundance of a genus of Enterobacteriaceae and a lower abundance of a family of Clostridiales during the symptomatic period. We saw some of these significant taxonomic differences even prior to symptom onset. There were no consistent longitudinal differences in richness or stability diversity metrics between infants with FPIAP and healthy controls.

**Conclusions:**

This study is the first to identify differences in the infant gut microbiome in children who develop FPIAP, some even before they develop symptoms, and provides a foundation for more mechanistic investigation into the pathogenesis of FPIAP and subsequent food allergic diseases in childhood.

Video abstract.

**Supplementary Information:**

The online version contains supplementary material available at 10.1186/s40168-022-01322-y.

## Introduction

Food protein-induced allergic proctocolitis (FPIAP) is a commonly recognized and burdensome form of non-IgE-mediated food allergy of early infancy with rates as high as 17% recently reported in the USA from the GMAP study [1]. FPIAP is typically diagnosed clinically by the presence of bloody and mucoid stools during the first few months of life in a generally healthy infant that resolves with dietary restriction (most commonly of milk protein and sometimes soy) [2]. FPIAP can be seen in both children who are breastfed and formula fed, but exclusively formula-fed infants are at increased risk [1]. The pathophysiology of FPIAP is not well studied or understood. It resolves for most affected patients within the first 12 months of life; however, it has been associated with an increased risk of developing both IgE-mediated food allergy (IgE-FA) [3] and eosinophilic esophagitis [4]. The rate of rise of food allergies strongly implicates environmental factors (antibiotics, diet, and other exposures) resulting in dysbiosis (microbial imbalance) which has been already associated with other forms of pediatric allergy [5, 6]. Complex cross talk between the intestinal microbiome, food antigens, intestinal inflammation, and the innate immune system early in life likely contributes to the mechanisms responsible for either healthy tolerance acquisition or food allergy development [7]. Given the early onset of FPIAP, association with feeding practices, and symptoms of the lower (and often also upper) GI tract, we hypothesized that the developing infant microbiome plays a role in FPIAP development and subsequent loss of oral tolerance.

There is a growing body of evidence that the gut microbiome plays a key role in the development of IgE- and non-IgE-mediated food allergy [8–15]. Infants with IgE-mediated cow’s milk allergy (IgE-CMA) have key taxonomic differences when compared to healthy infant counterparts [9, 12, 13]. They have additionally been shown to have taxonomic differences once started on extensively hydrolyzed formula and after achieving tolerance to milk protein, suggesting a role for the microbiome in disease resolution [12, 13, 16]. Literature on non-IgE-mediated cow’s milk protein allergy has similarly demonstrated taxonomic differences between healthy and allergic infants, infants on an extensively hydrolyzed formula diet, and infants tolerant to milk protein [8–11, 17, 18]. This literature, however, has been limited by cross-sectional design [8, 9], lack of strict diagnostic criteria for non-IgE-CMA [10, 11], detection methods by culture [10, 11], and pooling of non-IgE with IgE-CMA [9, 13]. To our knowledge, there is no study that has analyzed samples longitudinally including those collected prior to symptom or disease onset.

The Gastrointestinal Microbiome and Allergic Proctocolitis (GMAP) study is the first prospective observational healthy infant cohort study specifically designed to investigate the epidemiology, clinical presentation, and potential role of the longitudinal microbiome in FPIAP [1] and subsequent IgE-FA [3]. Over 1000 healthy infants were enrolled at their first pediatrics visit in the first week of life, stool samples were collected very frequently across the first year, and they are being followed clinically with ongoing sample collection through age 18. A total of 17% of these healthy children went on to develop FPIAP [1], and 6% went on to develop IgE-FA [3]. This large cohort addresses the many limitations in the literature, as we enrolled infants in an unbiased fashion, sampled infants frequently very early in life, and thus have sampling before, during, and after disease onset. This provided us with a well-matched control population, dense early sampling, and the ability to examine factors which may precede disease onset, herald symptom progression, or be associated with disease resolution.

## Results

### Participant profiles

**Fig. 1:**
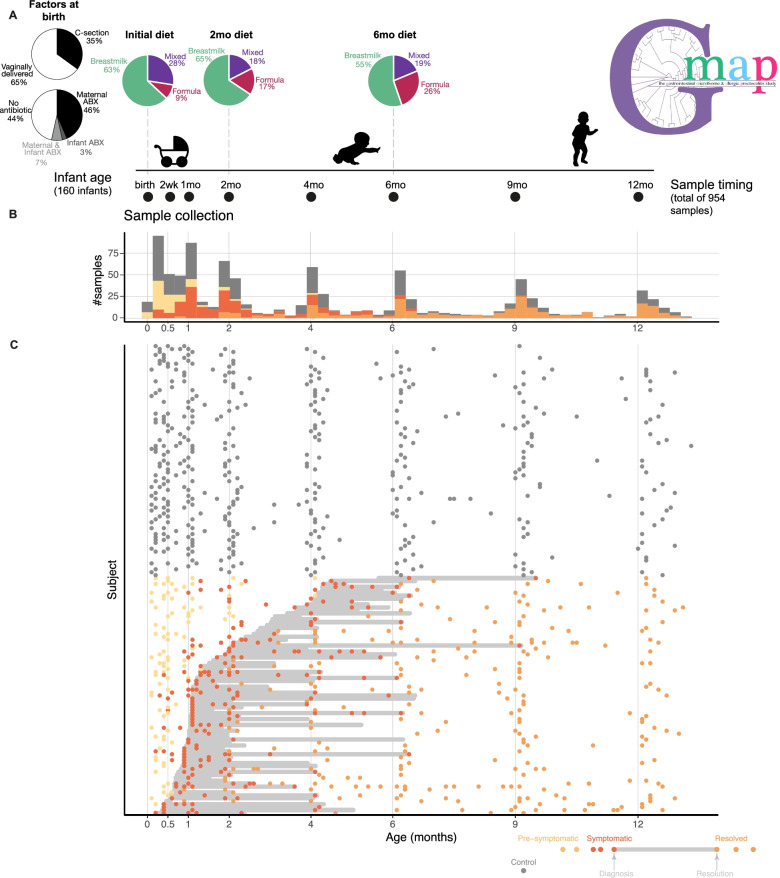
GMAP study cohort design, key clinical characteristics, and longitudinal sampling. **A** Key clinical and environmental features of the cohort analyzed including antibiotic exposure, mode of delivery, and infant diet over time and the timeline for sample collection. **B** Number of samples analyzed across the first year, binned by the age of the infant at the time of sample collection, and colored by whether that infant was a healthy control or a child with FPIAP. Samples from infants with FPIAP are colored by their symptom state at the time of sample collection. **C** Sample map showing the samples analyzed plotted by the age of the infant at time of collection and colored by their disease and symptom state. The horizontal light gray bars represent the time from diagnosis to resolution of symptoms

From the GMAP cohort [1], we selected the first 81 infants prospectively diagnosed with food protein-induced allergic proctocolitis (FPIAP) in the cohort with adequate longitudinal sampling and 79 matched controls with robust fecal microbiome sampling across their first year of life (Fig. 1, see also “Methods”). For infants who developed FPIAP, samples were selected from before symptom onset, during their symptomatic period, and after their symptoms resolved (Fig. 1). A total of 954 samples met quality control criteria (“Methods”) when sequenced using 16S ribosomal RNA gene sequencing. The median number of samples in the first year per child was 5 [7, 19]. Data were analyzed using QIIME2 (see “Methods”), generating a genus-level composition map for each sample. A total of 45% were female, 65% were delivered vaginally, 63% were initially exclusively breastfed, and 56% were perinatally exposed to antibiotics (maternal 46%, perinatal infant 3%, or both 7%) (Fig. 1). No significant differences among any of these factors were noted between infants with FPIAP and controls (Supplemental Table 1), with the exception of the initial infant diet which was more commonly formula in the children who developed FPIAP as has been previously reported [1].

### Overall microbiome composition

We first examined the microbiome collectively across all infants, looking for characteristic features of the developing infant gut microbiome in the first year of life. We found a predominance of *Bifidobacterium*, *Bacteroides*, Enterobacteriales, and *Clostridium* at the earliest time points as has been previously shown [19–22] (Supplemental Fig. 1A). We also saw the characteristic rise in overall microbial richness from birth to age 1 [19, 22, 23] (Supplemental Fig. 1B). Consistent with existing literature [24, 25], we found that infants who were vaginally delivered had a greater abundance of *Bacteroides* (*p* = 5.25 × 10^−7^, *q*-value = 5.06 × 10^−6^, coefficient = 0.23) compared to those delivered by C-section, and infants who were exclusively breastfed had a greater abundance of *Bifidobacterium* (*p* = 3.82 × 10^−4^, *q* = 2.25 × 10^−3^, *c* = 0.13) (Supplemental Fig. 1C). Additionally, infants who received any probiotics in the first year (predominantly *Lactobacillus rhamnosus* GG in this cohort) had a greater abundance of *Lactobacillus* (Supplemental Fig. 1C). Therefore, as we turned our attention to exploring taxonomic features that differentiated infants with FPIAP compared to healthy controls, we included age, mode of delivery, diet, and probiotic exposure in all of our multivariate linear models (using EasyMap, our newly developed interactive tool; “Methods”) reported here. Including perinatal antibiotic exposure in the model did not meaningfully change the results. Only results that met an absolute coefficient threshold of > 0.05 and *q*-value < 0.20 are reported (“Methods”). A complete table of all significant findings are available in Supplemental Table 2.

### Taxonomic differences between FPIAP cases and controls

**Fig. 2:**
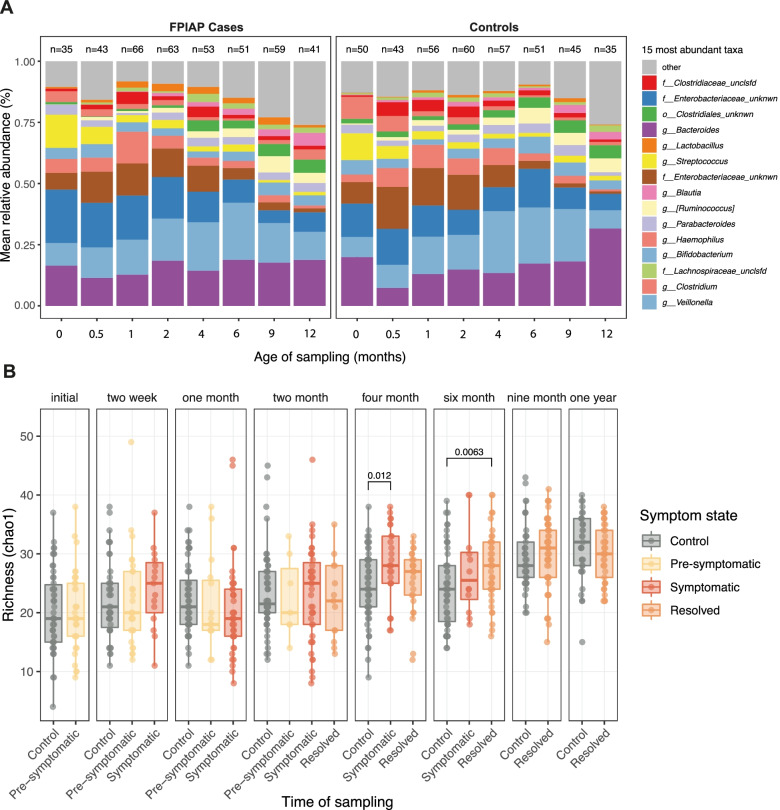
Longitudinal microbiome composition in infants with FPIAP compared to controls. **A** Composition plots showing the mean relative abundance of the top 15 abundant taxa and their longitudinal taxonomic assemblage over the first year in infants with FPIAP (left) compared to controls (right). **B** Alpha diversity measured by chao1 richness index in controls compared to infants with FPIAP before symptom onset, during the symptomatic period, and after symptom resolution over the first year (*p*-values calculated by *t*-test)

In examining potential differences in the infant microbiome in children who developed FPIAP compared to those who did not, we first looked for overall differences in community structure across the first year. While the overall first year composition was similar between cases and controls (Fig. 2A), we found a greater abundance of an unknown genus of Enterobacteriaceae (most likely, represented by *Escherichia*; “Methods”) in infants with FPIAP compared to controls. This genus of Enterobacteriaceae is also found in greater abundance in symptomatic FPIAP cases (*p* = 2.7 × 10^−2^, *q* = 1.13 × 10^−1^, c = 0.09) when analyzing all samples (Supplemental Table 2). We did not find any statistically significant differences between the overall microbial richness (Fig. 2B) or stability (Supplemental Fig. 2) across the first year when comparing FPIAP cases and controls, though some pairwise differences were noted at 4 and 6 months (between symptomatic cases and resolved cases respectively compared to controls, Fig. 2B).

Given that FPIAP presents so early in infancy (median age of diagnosis = 1 month, > 75% diagnosed by 2.5 months), we took two approaches to narrow our focus on samples from children at the times of most interest in this disease. First, we focused on all samples collected between 0 and 2 months of age (when the majority of cases of FPIAP have presented). Second, we made three key sample subsets: *last pre-symptomatic* (where for each case of FPIAP, we chose their last pre-symptomatic sample and then the nearest age-matched control), *first symptomatic* (where for each case of FPIAP, we chose their first symptomatic sample and then the nearest age-matched control), and *first resolved* (where for each case of FPIAP, we chose their first resolved sample and then the nearest age-matched control; Supplemental Fig. 3).

**Fig. 3:**
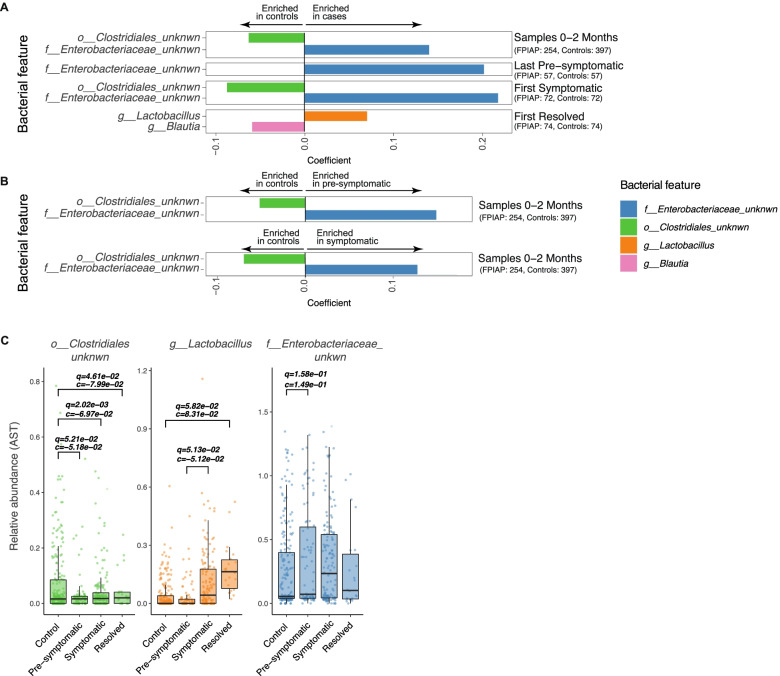
Summary of key differential taxa between infants with FPIAP and healthy controls. **A** Significantly different taxa comparing infants with FPIAP to healthy controls (*q* < 0.20; absolute coefficient > = 0.05) when looking at sample subsets: 0–2 months, last pre-symptomatic, first symptomatic, and first resolved. Bars to the right are enriched in infants with FPIAP, while bars to the left are enriched in the controls. Number of samples in each group is shown under the name of the subset analyzed in that model (FPIAP, control). **B** Significantly different taxa (*q* < 0.20) when comparing infants with FPIAP to matched controls before their symptom onset (top section) and then during the symptomatic period (lower section) over the first 2 months of age. Association directionality and numbers are as in (**A**). **C** Relative abundance trajectories of the key differential taxa between FPIAP cases and controls identified in **A** and **B** across symptom states (from pre-symptomatic to symptomatic to resolved). “q” indicates *q*-value and “c” indicates coefficient. Only significant *q*- and *c*-values are shown. Both values are directly generated from MaAsLin2 analysis. All models in this panel take into account all five variables: (a) case/control or symptoms, (b) mode of delivery, (c) age at visit, (d) diet, and (e) probiotics use in the first year of life

In the 0–2 months sample subset, we again found a greater abundance of the unknown genus of Enterobacteriaceae (*p* = 1.54 × 10^−2^, *q* = 0.11, *c* = 0.140) and a lower abundance of the unknown family of Clostridiaceae (*p* = 4.8 × 10^−5^, *q* = 1.27 × 10^−3^, c = −0.063) in FPIAP cases compared to controls (Fig. 3A).

### Taxonomic differences between FPIAP disease states and controls

We next looked for taxonomic differences that might differentiate phases of FPIAP development (pre-symptomatic, symptomatic, and resolved) from controls (“Methods”). Comparing infants with FPIAP before they developed symptoms (“pre-symptomatic”) to matched controls over the first 2 months of life, we again found a greater abundance of the same unknown genus of Enterobacteriaceae (*p* = 1.89 × 10^−2^, *q* = 0.16, *c* = 0.15) and a lower abundance of the unknown family of Clostridiales (*p* = 3.41 × 10^−3^, *q* = 5.21 × 10^−2^, *c* = −0.052) (Fig. 3B). Within the *last pre-symptomatic* subset, we saw the same enrichment of Enterobacteriaceae (*p* = 0.134, *q* = 0.192, *c* = 0.202) in infants with FPIAP (Fig. 3A). Next, we looked at the symptomatic FPIAP cases and found a greater abundance of the Enterobacteriaceae genus (*p* = 1.54 × 10^−2^, *q* = 1.13 × 10^−1^, *c* = 1.4 × 10^−1^) and a lower abundance of the family of Clostridiales (*p* = 4.8 × 10^−6^, *q* = 1.27 × 10^−3^, *c* = −6.33 × 10^−2^) in FPIAP cases compared to controls over the first 2 months. The same enrichment of the unknown genus of Enterobacteriaceae and lower abundance of the family of Clostridiales was seen in the *first symptomatic* sample subset (*p* = 2.05 × 10^−3^, *q* = 3.77 × 10^−2^, *c* = 0.217; *p* = 5.99 × 10^−6^, *q* = 5.5 × 10^−4^, *c* = −0.087, respectively) (Fig. 3A). Finally, in the *first resolved* sample subset, we found an enrichment of the genus *Lactobacillus* (*p* = 5.86 × 10^−3^, *q* = 7.17 × 10^−2^, *c* = 0.07) and a decreased abundance of the genus *Blautia* (*p* = 1.22 × 10^−2^, *q* = 0.105, *c* = −0.06) in infants with FPIAP which had resolved compared to controls (Fig. 3A).

Next, we were interested in the trajectories over time of each of these most significantly differential taxa as the infants’ disease progressed from pre-symptomatic, to symptomatic, to resolved states. Looking at all samples across the first year, the unknown family of the Clostridiales class was significantly lower in children with FPIAP, before they became symptomatic, while they had symptoms, and after they had resolved when compared to controls (Fig. 3C) with a relatively static trajectory. *Lactobacillus*, on the other hand, was lowest when infants were pre-symptomatic (comparable to controls), rose sharply as they became symptomatic, and then reached a significantly higher abundance than controls when infants’ symptoms had resolved (Fig. 3C). Infants with FPIAP were more likely to receive *Lactobacillus*-containing probiotics, so we next examined whether this could explain the differential abundance of *Lactobacillus*. Stratifying by probiotic exposure in the first year, we found that while indeed infants receiving probiotics had a higher abundance of *Lactobacillus*, the differential trajectory was seen in both groups, regardless of probiotic use (Supplemental Fig. 4). The unknown genus of Enterobacteriaceae also has an interesting trajectory: peaking during the symptomatic period (there is a nonsignificant higher relative abundance than controls even in the pre-symptomatic group and then abundance rises significantly higher than controls as infants become symptomatic and then decreases but remaining nonsignificantly higher than controls even once symptoms have resolved (Fig. 3C).

### Machine learning approach for predicting disease state from microbial community

**Fig. 4:**
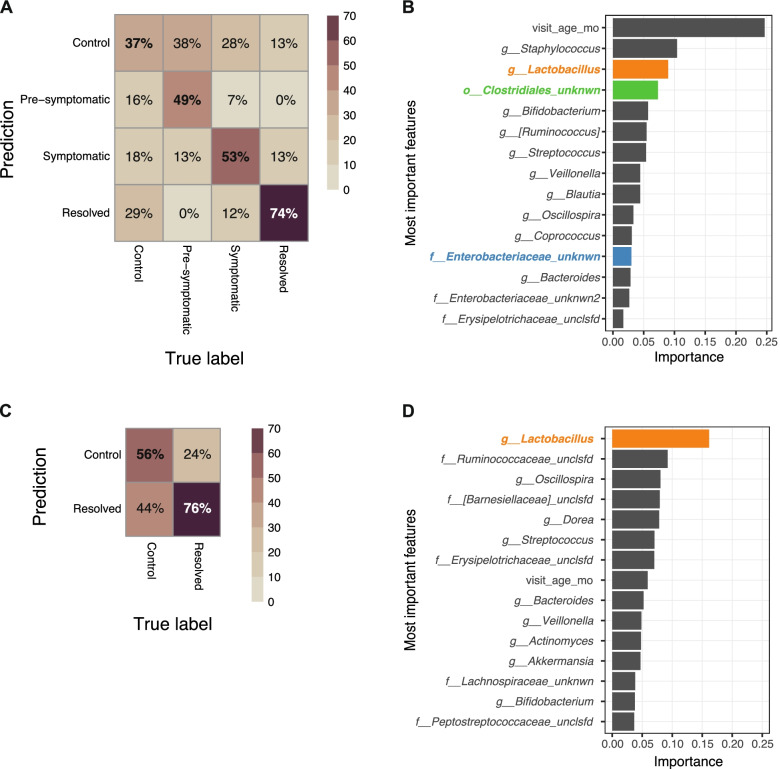
Independent machine learning approach identifies similar differential taxa between infants with FPIAP and healthy controls. **A**–**C** Random forest prediction accuracy across all samples (**A**) and in those over 6 months comparing controls with resolved FPIAP cases (**C**). Most important features identified in differentiating disease states across all samples (**B**) and those over 6 months (**D**). Colored taxa in **B** and **D** represent those also independently identified in our multivariate analyses shown in Fig. 3

Finally, we applied an independent machine learning algorithm to try to distinguish between the microbial communities across the disease states and compared to control samples. Instead of searching for individual taxonomic features that are differential, the random forest approach incorporates information from multiple features simultaneously to predict the disease state. We estimated our success by examining the prediction accuracy in each of the disease states, and we found that overall, the true classification is the most probable one (Fig. 4A). When examining the importance of specific features in the accuracy of the prediction, we find some novel features and also confirm some of the taxonomic features also identified by the multivariate analysis (Fig. 4B). The most recurring findings in all models were the unknown family of Clostridiales, and the unknown genus of Enterobacteriaceae, which were further validated here by being independently identified in this orthogonal analytic approach.

Next, we observed that our model correctly classified the resolved samples with particularly high probability (74%). We wanted to identify the features that enable this specific separation. Towards this end, we focused on a simpler scenario, using samples from age 6 months and older (when most samples are either from a control or resolved). Running the random forest model on this smaller subset of samples (133 control and 182 resolved) was as successful in correctly classifying the resolved samples (76%; Fig. 4C). The most important feature in this classification was found to be *Lactobacillus* abundance (as in the multivariate model above), and interestingly, the sample age did not play as an important role as in the null model (Fig. 4D).

## Discussion

Food protein-induced allergic proctocolitis (FPIAP) is often the earliest manifestation of food allergy in children, and yet little is known about its pathogenesis. Few studies have examined the microbiome of infants with FPIAP, and to our knowledge, none has dense longitudinal sampling which includes samples prior to symptom onset. From the GMAP study, we performed 16S rRNA gene sequencing on 954 longitudinal samples from 160 infants (81 with FPIAP and 79 matched controls) from 1 week to 1 year of age. While overall the composition and richness of the infants’ microbiome was similar in infants with FPIAP compared to controls, we identified several taxonomic differences in infants with FPIAP across symptom states (from pre-symptomatic to resolved). Importantly, some differential features were seen before symptom onset and after symptom resolution, suggesting that these features are not simply a reflection of the inflammatory disease state.

We found an increased abundance of an unknown genus of the Enterobacteriaceae family which was most prominent when infants were symptomatic with FPIAP but was elevated even prior to symptom onset. The Enterobacteriaceae family contains species known to bloom in other inflammatory settings in the GI tract [26] and has previously been associated with cow’s milk allergy [13, 16] and other food allergies [27]. We also found a decreased abundance of an unknown family of Clostridiales. Some families within this order (Peptostreptococcaceae, Lachnospiraceae, some *Clostridium* species) have been previously associated with decreased relative abundance in children with IgE- and non IgE-mediated food allergies [9, 28, 29], although many other Clostridiales species are enriched. Previous reports suggest that specific species of Clostridia drive the separation between allergic children and their nonallergic siblings and controls [28]. Among these families are species which are important for gut permeability/integrity as well as butyrate production, both of which have important potential mechanistic roles in FPIAP warranting further study [8, 30]. We also found a greater abundance of members of the genus *Lactobacillus* as infants with FPIAP’s symptoms resolved compared to controls (irrespective of probiotic use). Other studies of infants with a mix of IgE- and non-IgE-mediated cow’s milk allergy have shown an increase in *Lactobacillus* when infants were treated with hypoallergenic formula [13], FMT [31], or hypoallergenic formula + LGG [8]. Finally, we found an unknown family of the Clostridiaceae family which was significantly lower across symptom states in these allergic infants. A few IgE-FA studies have also shown decreased Clostridia species [16, 32] in food allergic subjects.

One limitation inherent to studying the microbiome of FPIAP is how early in infancy the disease presents. The microbiome composition in the first few months of life is highly dynamic, making it more difficult to detect differences between clinical groups. This likely accounts for some of our findings which appeared in some sample subsets but not others but also underscores the significance of those findings that were replicated in several subsets and across time. The limited number of samples in our pre-symptomatic sample subset, due to very young age, also likely limited our power to identify differences in that subset. Another challenge to understanding FPIAP pathogenesis with respect to the microbiome is that the diet often changes as infants are diagnosed (some with maternal dietary elimination, few with changes from breastmilk to formula, and some with changes to different formula types) which can have a significant impact on the microbiome and cannot be fully accounted for in these models. This is why we focused many of our analyses on subsets prior to or at the time of disease onset (before dietary changes would have been made). There are a few other limitations to our study. We were not able to adequately capture, and therefore control for, infectious episodes or infant antibiotic use at the time of each sample. However, including perinatal antibiotic exposure in our model did not meaningfully change our results. Additionally, the time of resolution of FPIAP was not prospectively systematically assessed and so was estimated based on parent report and chart review as previously published [1].

## Conclusions

In summary, our unique longitudinal pediatric cohort enabled us to carefully investigate the role of the microbiome in one of the earliest manifestations of allergy in children (FPIAP). Because of the prospective healthy infant cohort design, we were able to identify key taxonomic differences in children who developed FPIAP, even before they became symptomatic, as well as after their disease resolved. We were also able to contribute to the growing body of literature describing the early composition of the infant microbiome in nonallergic children and highlight several potential areas of important further investigation. Our study provides a foundation for testable hypotheses around the underlying role of potentially key taxa including Enterobacteriaceae and Clostridiales in the pathophysiology of FPIAP. It remains important to further determine these mechanisms as they may provide novel opportunities for early targeted interventions to prevent IgE and non-IgE-mediated food allergy more broadly and perhaps to better support an optimal pediatric microbiome promoting healthier nonallergic phenotypes.

## Methods

### Patient characteristics

From the GMAP prospective observational healthy infant cohort, we selected the first 81 infants diagnosed with food protein-induced allergic proctocolitis (FPIAP) who had a minimum of 4 longitudinal stool samples in the first year. FPIAP was diagnosed by the treating physician and confirmed by comprehensive study staff chart review. Prespecified case inclusion criteria included guaiac positive or grossly bloody stool as previously published [1]. We then selected all possible controls who met the same sampling criteria and volume and from those randomly selected controls at 1:1 ratio. We confirmed by testing equality of proportions that the selected nested case-control cohort for this microbiome study matched the reported GMAP cohort at large with respect to key covariates including mode of delivery, perinatal antibiotic exposure, and initial infant diet [1].

### Sample collection

Fresh diapers were brought to the clinic visit, where stool samples were collected by research study staff into sterile tubes using established protocols, stored immediately at −20 °C, and then transferred to −80 °C for storage until processing.

### DNA extraction and sequencing

We used the Qiagen DNeasy PowerSoil htp 96 (Cat no./ID: 12955-4) to extract stool samples that were stored at −80 °C and “chipped” while frozen to obtain 100–150 mg of sample for extraction. Bead beating was performed on a TissueLyser at 20 Hz for 10 min as per manufacturer’s protocol.

16S rRNA gene libraries targeting the V4 region of the 16S rRNA gene were prepared by first using qPCR to normalize template concentrations and determine optimal cycle number. Library construction was performed in quadruplicate with the primers 515F (5′-AATGATACGGCGACCACCGAGATCTACACTATGGTAATTGTGTGCCAGCMGCCGCGGTAA-3′) and unique reverse barcode primers from the Golay primer set [33]. After amplification, sample replicates were pooled and cleaned via Agencourt AMPure XP-PCR purification system. Prior to final pooling, purified libraries were normalized via qPCR in two 25-uL reactions, 2× iQ SYBR SUPERMix (Bio-Rad, REF: 1708880) with Read 1 (5′-TATGGTAATT GT GTGYCAGCMGCCGCGGTAA-3′), Read 2 (5′-AGTCAGTCAG CC GGACTACNVGGGTWTCTAAT-3′) primers. Pools were quantified by Qubit (Life Technologies, Inc.) and sequenced on an Illumina MiSeq 300 using custom index 5′-ATTAGAWACCCBDGTAGTCC GG CTGACTGACT-3′ and custom Read 1 and Read 2 primers mentioned above. Raw sequencing data can be found on NCBI BioProject PRJNA730851.

### 16S rRNA gene sequencing analysis

Microbial communities were analyzed using QIIME2 version 2020.11.1 [34]. The DADA2 algorithm [35] was used for quality filtering and merging sequences with greater than 99% similarity. Alignment was done by use of “qiime feature-classifier fit-classifier-naive-bayes.” A total of 1088 samples were collected. Samples that did not pass the sequencing process had a total frequency of less than 3000 reads, or those with an unclear diagnosis were dropped. In addition to the DADA2 filtering process, features represented in only one sample or with a maximal relative abundance of < 0.03 were also removed. A total of 954 samples remained for analysis. Relative abundance data were normalized with QIIME2’s tools. A reference Greengenes database version 13.5 [36] was used to assign taxonomy features.

OTUs that were *unclassified* on a certain taxonomic level were assigned to be an “unclassified taxa” from the level above. For example, some OTUs that were not classified on a family level got an “unclassified” assignment on the order level. For one of these sets that had significant results in our data, we attempted to manually identify a better classification. We used blast-2.10.0+ to map the OTU sequences against the NCBI nt database (downloaded on May 16, 2017). Our results suggest that the unknown genus of the Enterobacteriaceae family is most likely representing sequences from the *Escherichia* genus.

### Statistical analysis

All analyses were performed in RStudio (Version 1.3.1093) run with R (version 4.0.2). Data editing was done with dplyr. Graphs were created using R packages ggplot2 and ggpubr. Alpha diversity was calculated using the fossil package. Multivariate regression analysis was performed using the MaAsLin2 R package. Mixed-effects linear models using a variance-stabilizing arcsin square root transform (AST) on relative abundances are then used to determine the significance of putative associations from among this reduced set. The AST function is defined as follows:

$$AST(x):= \operatorname{sign}(x)\cdot \arcsin \kern0.5em \left(\sqrt{\left|x\right|}\right)$$

It is a monotonic function that, in our case, maps values from [0, 1] to [0, 1.57], such that zero remains at zero, AST(0) = 0, and then spreads the rest. Its intuition is similar to a log transformation which is commonly used to spread the data, only in the AST zero values are maintained, unlike in the log transformation where log(0) is not defined. It was originally described to deal with proportional data, such as microbiome relative abundance [37]. Nominal *p*-values across all associations were then adjusted using the Benjamini–Hochberg FDR method. An FDR < 0.25 is the standard default using MaAsLin2. Any taxon-level association with an FDR-corrected *q*-value < 0.25 is commonly considered statistically significant in microbiome studies where further investigation and validation of results are necessary [38–40]. We elected to use a more strict threshold and report here only associations with an *FDR* < 0.2. *p*-values that did not originate from the MaAsLin analysis were generated using the rstatix R package.

All boxplots were generated using ggplot2: The lower and upper hinges correspond to the first and third quartiles (the 25th and 75th percentiles), and the upper and lower whiskers roughly represent the 1.5 × IQR from the hinge (default geom_boxplot function).

The model coefficient values (named also effect size) indicate the amplitude of the change between one category values to the reference category value. A positive coefficient indicates a positive correlation, and a negative value indicates a negative correlation between the independent and the dependent variables.

### Composition and richness analysis

The infant gut microbiome richness greatly increases during the first year of life [41, 42]; thus, we performed the richness analysis per age group. We split the 954 samples to eight time points (0, 0.5, 1, 2, 4, 6, 9, 12) and labeled each sample with its closest time point. Some subjects had multiple samples that were mapped to the same age group; thus, we kept only a single sample per subject in each group (we chose the sample with the closest time to the age group). Following this step, we were left with 808 samples, divided into eight age groups. Richness was calculated with the chao1 function in the fossil R package.

For the stability analysis, we used the same subset of 808 samples (described above) and calculated microbial composition stability in all pairs of consecutive samples. We used the Bray-Curtis and Jaccard function to calculate this measure.

For the composition analysis, we calculated the average relative abundance of each microbial feature in each age group and then normalized this distribution to sum up to 1 (Fig. 2A, Supplemental Fig. 1A).

### Multivariate models definition

To find a linear correlation between FPIAP and microbial features, we used three different models: (1) comparing samples from FPIAP cases to controls; (2) comparing samples based on their FPIAP disease state (pre-symptomatic, symptomatic, and resolved) to controls; and (3) as in (2) but comparing the samples to the symptomatic group as a reference. Each model contains the following fixed effects variables: (a) case/control or symptoms, (b) mode of delivery, (c) age at visit, (d) diet, and (e) probiotics use in the first year of life. As we have multiple samples from each child, all models also include the child ID as a random effect variable.

In general, we ran the models on six different subsets of samples, either subsetting by age or by disease status:

Age subgroups are as follows:

- All samples — This group includes all samples (*n* = 954).
- Zero to two months — All samples with age visit < 3 months (*n* = 473)
- Six-plus months — All samples with age visit in the range 6.1–13.3 months (*n* = 315)

Disease status subgroups (these subsets always contain a single sample per subject) are as follows:

d. *Last pre-symptomatic* — For each FPIAP case, we chose the last sample collected prior to symptom onset and identified a matched control sample (*n* = 114).
e. *First symptomatic* — For each FPIAP case, we chose the first sample when the child was symptomatic and identified a matched control sample (*n* = 144).
f. *First resolved* — For each FPIAP case, we chose the first sample when the child was asymptomatic and identified a matched control sample (*n* = 148).

For the disease status subgroups analyses, we used the Hungarian algorithm to randomly select the set of control samples that were closest in age to the FPIAP samples in those subsets. These selections are shown in Supplementary Fig. 3. Matched control samples were chosen by finding an optimal match between the FPIAP sample times and all the control samples, making sure to include at most a single sample from each control subject in each subset. We found the optimal match by defining it as a linear sum assignment problem (LSAP) using the clue R package to solve it with the Hungarian method.

### Using the EasyMap tool

To run and compare the 1–3 models across the different subgroups, we used the EasyMap tool we developed in our lab [43], which enables rapid interactive multivariate linear regression analysis on microbiome data such as in here. The tool itself is available at https://yassour.rcs.huji.ac.il/EasyMap.

### Random forest analysis

Each sample was assigned a label based on the disease state: control, pre-symptomatic, symptomatic, or resolved. We constructed a random forest model with 78 microbial features and the sample age as an additional feature. We ran the model 100 times, wherein each iteration we randomly divided our data to 80% train and 20% test sets (763 and 190 samples, respectively).

To get the optimal random forest model, we ran a random search on multiple parameters, and we used the model that yielded the best result on a test set. The model has 1800 estimators, a max depth of 100, a minimum leaf size of 2, and a minimum split size of 5. Our dataset contains 43% control samples, 10% pre-symptomatic samples, 21% symptomatic samples, and 25% resolved samples. To solve the imbalance in our data, we added class weights where we assigned: control, pre-symptomatic, symptomatic, and resolved classes of the weights 0.111, 0.444, 0.222, and 0.222, respectively. We have found that adding the age of the infant as a feature to the random forest improves the test accuracy by ~15%, and the age feature received the highest importance score over all other features.

Then, using the 6+ months subset, we selected only samples from age 6 months or older, and either a control or had resolved FPIAP. We ran a random search and chose the model with 1065 estimators, max depth of 5, a minimum leaf size of 2, and a minimum split size of 20. We have assigned the control and resolved classes the weights 1 and 0.9, respectively, to overcome the small imbalance in the data. Sample size was too small to stratify the random forest analysis by probiotic use.

We use the scikit-learn package in python to run the random forest models and calculate accuracy and importance for the trained models [44]. The importance score was calculated using the “Gini importance” which is defined as the sum over the number of splits that include the feature, proportional to the number of samples it split, and then summed and averaged across all trees. We present the normalized score for the top 15 features.

## Supplementary Information

**Additional file 1: Supplemental Figure 1**. Overall longitudinal microbiome composition. (A) Composition plot of the full first year of life showing the mean relative abundance of the top 15 taxa and their longitudinal taxonomic assemblage over the first year. (B) Longitudinal microbial richness (using the chao1 index) over the first year. The center line denotes the median, the boxes cover the 25th to 75th percentiles. (C) Relative abundance (arcsine transformed, AST) of key taxonomic differences mediated by important environmental factors: *Bacteroides* by delivery mode, *Bifidobacterium* by infant diet, and *Lactobacillus* by probiotic use. FDR-corrected *q*-values and coefficients are calculated from the multivariate analysis across all samples. **Supplemental Figure 2**. Community stability analysis calculated by Bray-Curtis beta diversity method for all consecutive sample pairs from the same subject. Each dot represents a sample pair, and is colored by the disease state of the first sample in the pair (*p*-values were calculated using a two sided t-test). **Supplemental Figure 3**. Sample subsets. (A) Flow diagram showing sample subsets used for analyses with their corresponding sample sizes and rationale. (B) A sample map showing longitudinal samples used for each subset analyzed, axes and colors as in Fig. 1. The horizontal light gray bars represent the time from diagnosis to resolution of symptoms. The ‘+’ sign represents samples that were not selected in any model. **Supplemental Figure 4**. Differential trajectory of *Lactobacillus* across disease states in infants with FPIAP compared to controls, stratified by probiotic use. Box plots of the relative abundance (AST) trajectories of *Lactobacillus* across disease states (from pre-symptomatic to symptomatic to resolved) compared to controls, stratified by (largely LGG-containing) probiotic use across all samples (*p*-values calculated using t-test).

**Additional file 2: Supplemental Table 1**. Demographics. Demographics of the infants from the GMAP cohort selected for this nested case-control microbiome study.

**Additional file 3: Supplemental Table 2**. Summary table of all significant MaAsLin results. All MaAsLin results from 16S rRNA gene sequencing with *q*-values for significant taxa which met our predetermined threshold for significance (q<0.20). Mixed effects linear models using arcsine transform on relative abundances were used to determine significance. *p*-values were adjusted for multiple comparisons using the Benjamini-Hochberg false discovery rate (FDR) method with FDR set at <0.20. (XLS 224 kb)

## Data Availability

Raw sequencing data can be found on NCBI BioProject PRJNA730851.
