# Supplementary figures and images for: Longitudinal disease-associated gut microbiome differences in infants with food protein-induced allergic proctocolitis
Source: Microbiome. 2022 Sep 23;10:154. doi: 10.1186/s40168-022-01322-y (PMC9503280; doi:10.1186/s40168-022-01322-y)

# Supplemental Figure 1

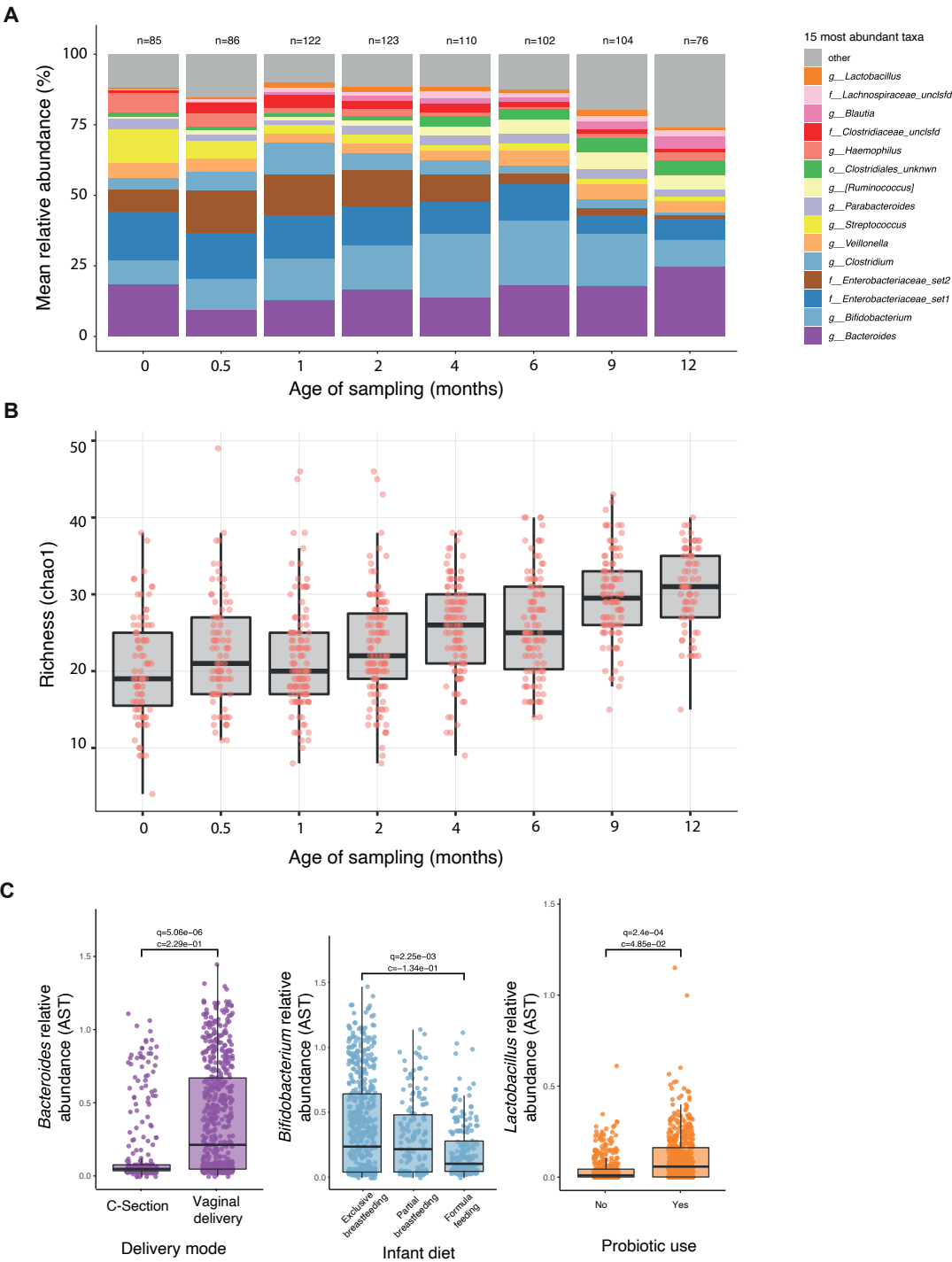

## Supplemental Figure 2

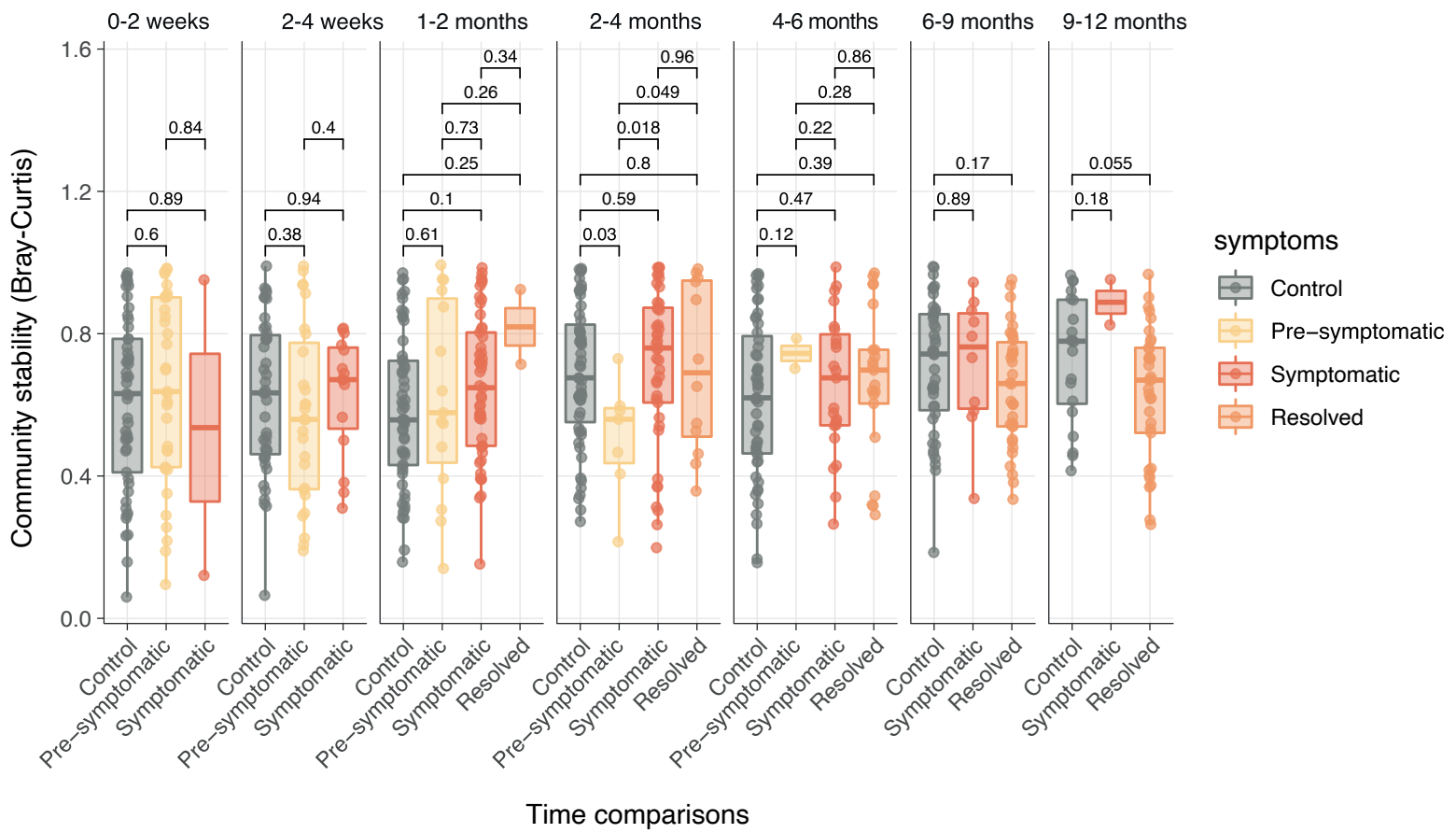

Supplemental Figure 3

A

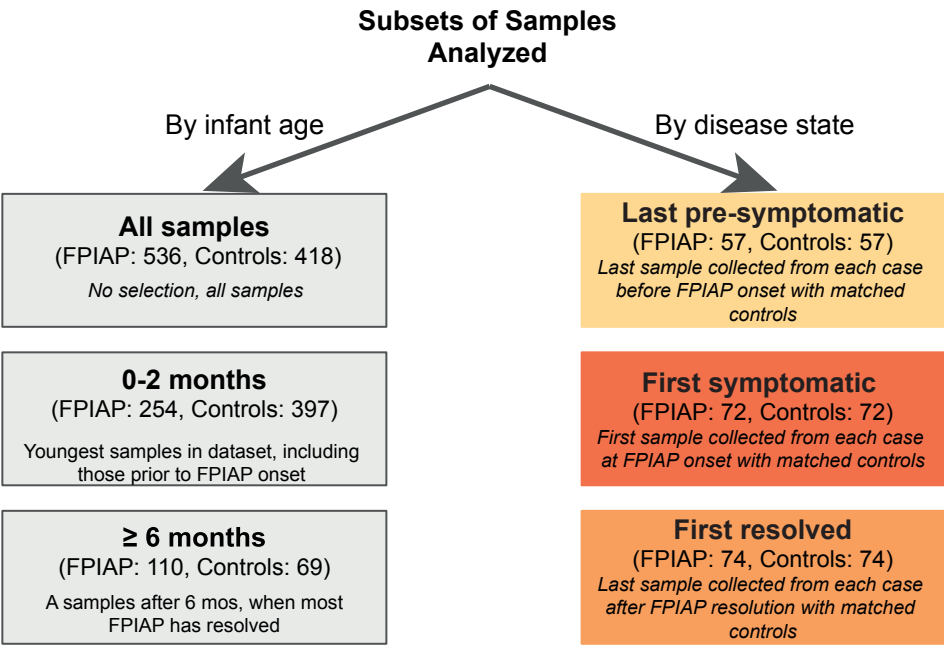

Supplemental Figure 4

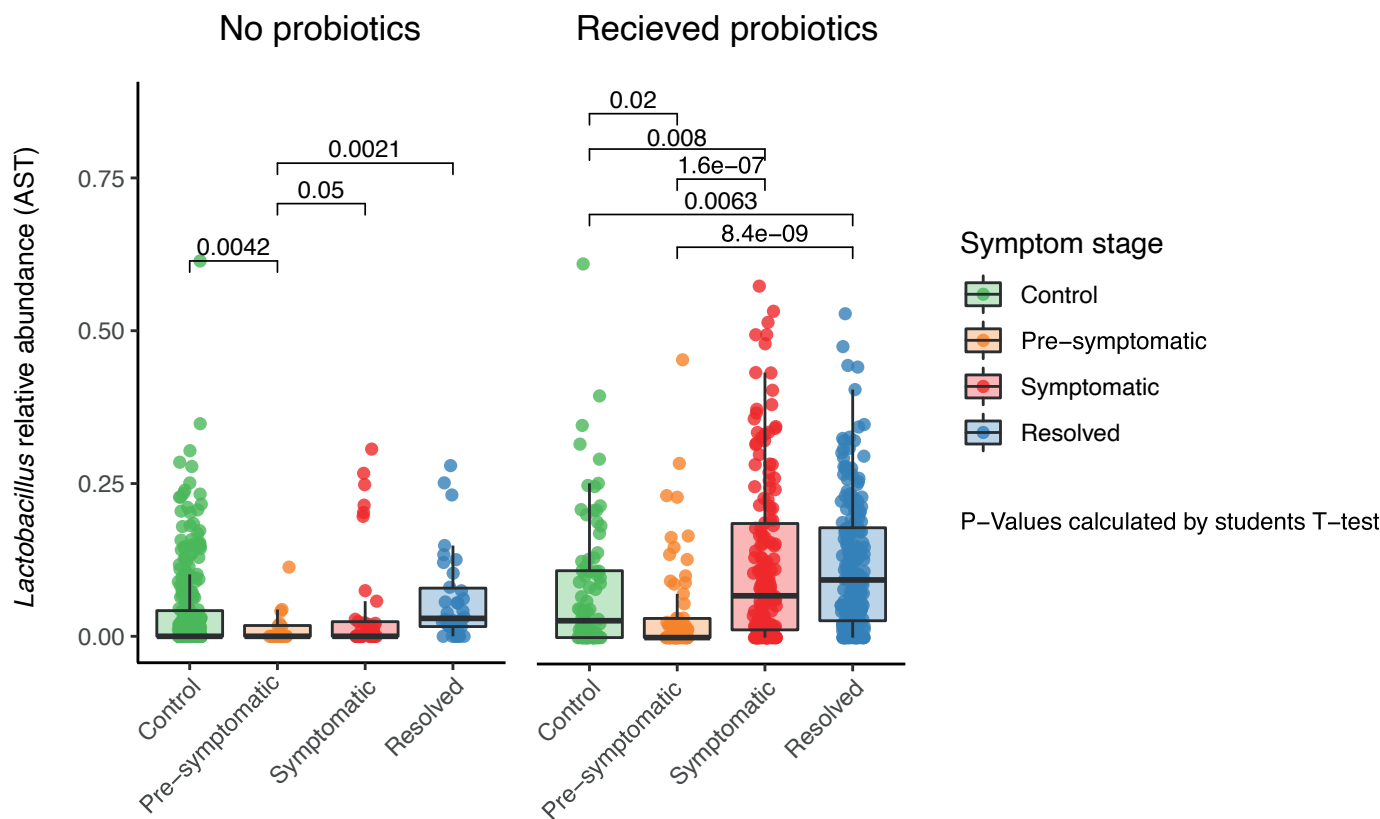

Supplement: Supplementary file 2 — Additional file 1: Supplemental Figure 1. Overall longitudinal microbiome composition. (A) Composition plot of the full first year of life showing the mean relative abundance of the top 15 taxa and their longitudinal taxonomic assemblage over the first year. (B) Longitudinal microbial richness (using the chao1 index) over the first year. The center line denotes the median, the boxes cover the 25th to 75th percentiles. (C) Relative abundance (arcsine transformed, AST) of key taxonomic differences mediated by important environmental factors: Bacteroides by delivery mode, Bifidobacterium by infant diet, and Lactobacillus by probiotic use. FDR-corrected q-values and coefficients are calculated from the multivariate analysis across all samples. Supplemental Figure 2. Community stability analysis calculated by Bray-Curtis beta diversity method for all consecutive sample pairs from the same subject. Each dot represents a sample pair, and is colored by the disease state of the first sample in the pair (p-values were calculated using a two sided t-test). Supplemental Figure 3. Sample subsets. (A) Flow diagram showing sample subsets used for analyses with their corresponding sample sizes and rationale. (B) A sample map showing longitudinal samples used for each subset analyzed, axes and colors as in Fig. 1. The horizontal light gray bars represent the time from diagnosis to resolution of symptoms. The ‘+’ sign represents samples that were not selected in any model. Supplemental Figure 4. Differential trajectory of Lactobacillus across disease states in infants with FPIAP compared to controls, stratified by probiotic use. Box plots of the relative abundance (AST) trajectories of Lactobacillus across disease states (from pre-symptomatic to symptomatic to resolved) compared to controls, stratified by (largely LGG-containing) probiotic use across all samples (p-values calculated using t-test). [file 40168_2022_1322_MOESM1_ESM.pdf]
