# Supplementary material for: Longitudinal disease-associated gut microbiome differences in infants with food protein-induced allergic proctocolitis
Source: Microbiome. 2022 Sep 23;10:154. doi: 10.1186/s40168-022-01322-y (PMC9503280; doi:10.1186/s40168-022-01322-y)
Supplement: Supplementary file 3 — Additional file 2: Supplemental Table 1. Demographics. Demographics of the infants from the GMAP cohort selected for this nested case-control microbiome study. [file 40168_2022_1322_MOESM2_ESM.pdf]

Characteristic	Overall<br>160 (100%)	Allergy status<br>FPIAP<br>81 (51%)	 Control<br>79 (49%)	p-value[1]
Female	70 (44%)	38 (47%)	32 (41%)	0.4
Race				0.3
Asian	32 (20%)	19 (24%)	13 (17%)	
Black	2 (1%)	1 (1%)	1 (1%)	
White	105 (66%)	54 (68%)	51 (65%)	
Multiple Race	17 (11%)	6 (8%)	11 (14%)	
Other	2 (1%)	0 (0%)	2 (3%)	
Hispanic or Latino	7 (6%)	4 (6%)	3 (5%)	>0.9
Vaginal Delivery	104 (65%)	55 (68%)	49 (62%)	0.4
Any Perinatal antibiotics	90 (56%)	45 (56%)	45 (57%)	0.9
Initial diet				0.3
Breastmilk	101 (63%)	52 (64%)	49 (62%)	
Mixed	44 (28%)	19 (23%)	25 (32%)	
Formula	15 (9%)	10 (12%)	5 (6%)	

[1] Pearson's Chi-squared test; Fisher's exact test
